# Inheritance of Telomere Length in a Bird

**DOI:** 10.1371/journal.pone.0017199

**Published:** 2011-02-22

**Authors:** Thorsten Horn, Bruce C. Robertson, Margaret Will, Daryl K. Eason, Graeme P. Elliott, Neil J. Gemmell

**Affiliations:** 1 School of Biological Sciences, University of Canterbury, Christchurch, New Zealand; 2 Department of Zoology, University of Otago, Dunedin, New Zealand; 3 National Kakapo Team, Department of Conservation, Nelson, New Zealand; 4 Centre for Reproduction and Genomics, Department of Anatomy & Structural Biology, University of Otago, Dunedin, New Zealand; Vrije Universiteit Medical Center and Center for Neurogenomics and Cognitive Research, Netherlands

## Abstract

Telomere dynamics are intensively studied in human ageing research and epidemiology, with many correlations reported between telomere length and age-related diseases, cancer and death. While telomere length is influenced by environmental factors there is also good evidence for a strong heritable component. In human, the mode of telomere length inheritance appears to be paternal and telomere length differs between sexes, with females having longer telomeres than males. Genetic factors, e.g. sex chromosomal inactivation, and non-genetic factors, e.g. antioxidant properties of oestrogen, have been suggested as possible explanations for these sex-specific telomere inheritance and telomere length differences. To test the influence of sex chromosomes on telomere length, we investigated inheritance and sex-specificity of telomere length in a bird species, the kakapo (*Strigops habroptilus*), in which females are the heterogametic sex (ZW) and males are the homogametic (ZZ) sex. We found that, contrary to findings in humans, telomere length was maternally inherited and also longer in males. These results argue against an effect of sex hormones on telomere length and suggest that factors associated with heterogamy may play a role in telomere inheritance and sex-specific differences in telomere length.

## Introduction

Telomeres are the ends of the chromosomes and consist of a repetitive DNA sequence [1] and a protein complex called shelterin [2]. Telomeres shorten with every cell division in somatic tissues of humans (and many other species) due to the end replication problem and other factors [reviewed in 3]. In addition to functions in cellular maintenance and replication [3], telomeres are also thought to be a defense mechanism against cancer, as short telomeres induce senescence and thus inhibit uncontrolled cell proliferation [4]. However, senescent cells also decrease the regeneration capacity of tissues, which can result in the onset of age-related diseases [5], [6]. Telomere length (TL), therefore, has been widely investigated as a marker for ageing and somatic fitness, as well as cancer in humans [4], [7].

Telomere length change has been associated with a growing list of environmental factors [4], but there is also a strong genetic component that influences TL in human and presumably other species [5], [6], [8]. Although contradictory modes of TL inheritance have been reported in humans [reviewed in 5], most studies support a pattern of paternal inheritance [9]–[11]. As inheritance of TL from father to daughter and son cannot be explained by heterosomal genes, Njajou et al [9] suggested that a paternal imprinting mechanism may deactivate one of the parental alleles of autosomal genes associated with TL regulation. In addition, woman generally have longer telomeres than men [5], [6] but it is not yet clear what causes this difference. Aviv [4] suggested that either the antioxidant properties of the female sex hormone oestrogen might result in a lower rate of telomere loss in females or a stochastic inactivation of one of the X-chromosomes.

As telomere structure and the proteins involved in TL regulation are highly conserved among vertebrates [1], we expect similar genetic and non-genetic factors to be present in most vertebrate species. To investigate the role of sex chromosomes in TL inheritance and regulation, we examined TL in a bird species because birds possess a ZW sex determination system in which females are the heterogametic sex in contrast to the XY sex determination system in humans in which males are the heterogametic sex. Since birds share non-genetic factors, including oestrogens, with humans, longer telomeres should also be evident in female birds if these are responsible for the difference in TL between sexes. Likewise if heterosomal genes or other factors linked to heterogamy (e.g. parental imprinting) influence TL inheritance, then the inheritance pattern in birds should be primarily maternal, opposite to that found in humans.

We chose to measure TL in an endemic New Zealand parrot, the kakapo (*Strigops habroptilus*). Although kakapo is not a classical avian model and the population is small, it possesses several characteristics that make it desirable for a comparative investigation of TL inheritance with humans. First, kakapo are very long lived: the oldest bird has a minimum age of 35 years, and some authors believe that he might even be close to 100 years old [12]. Thus, the life expectancy of kakapo is more similar to humans than that of other birds. Second, kakapo are extensively managed. As a result, kakapo receive health care, supplemental feeding and human assistance/monitoring of breeding making the level of intervention this species receives more akin to humans than most other bird species/populations. This is important, as a variety of environmental factors have been found to influence TL in birds [13] and might mask the effects of TL inheritance in wild or low managed species/populations. Last, active monitoring of breeding during the last 15 years and genetic screening of the entire population [14], [15] has produced a pedigree documenting parent-offspring relationships.

## Results

**Figure 1:**
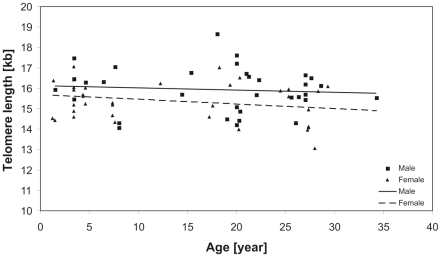
Telomere length of Kakapo against age. Solid line indicates regression of male TL and dotted line of female TL.

**Figure 2:**
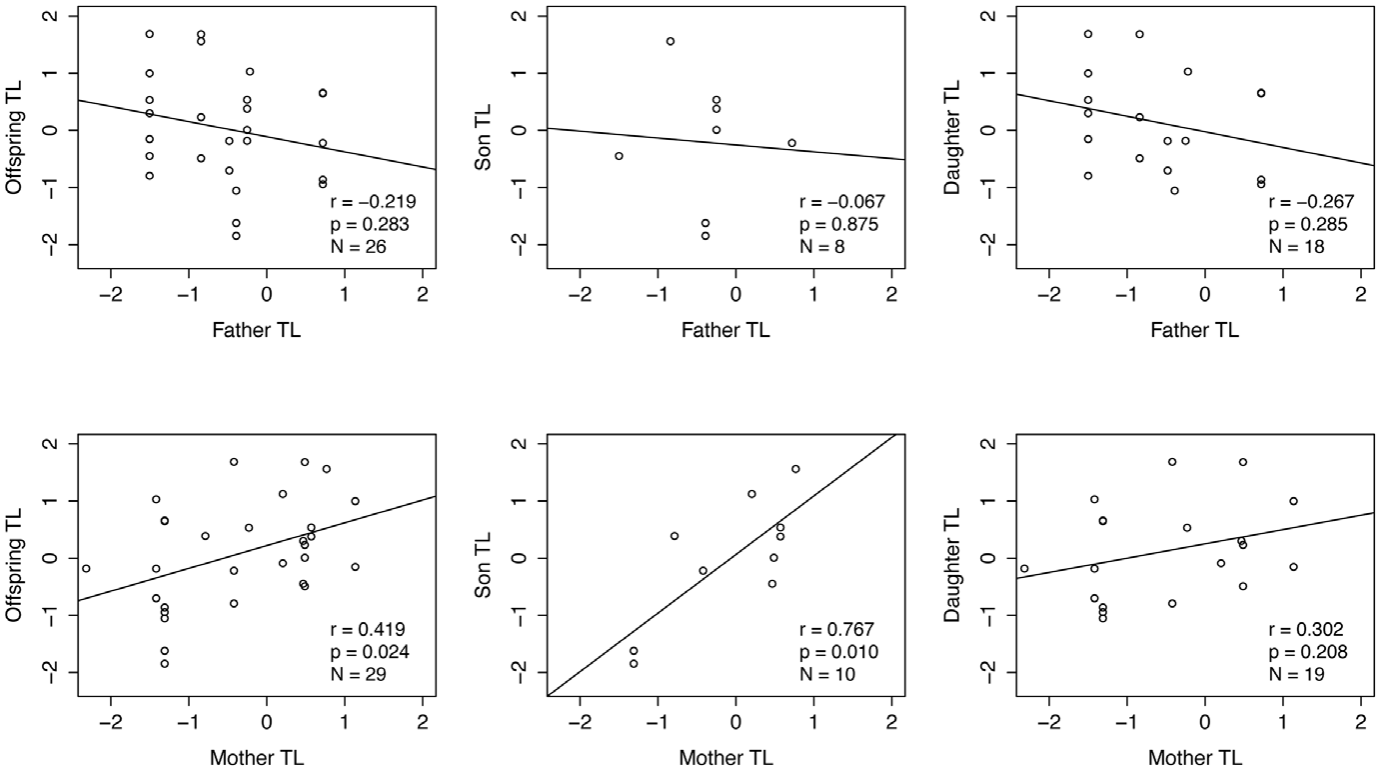
Parent-offspring correlations of kakapo telomere length corrected for sex.

TL ranged from 13.07 to 18.66 kb with a mean 15.64±1.06 kb across all samples. TL was shorter in females than in males (15.38±0.97 kb vs. 15.93±1.09 kb, t_67_ = 2.2046, p  =  0.031, Figure 1). Age was not correlated with telomere length before and after correcting for sex specific telomere length. Offspring TL was not correlated with parental or paternal TL (Table 1). Maternal TL was significantly correlated with TLs of offspring and of sons (Table 1, Figure 2).

**Table 1:** Parent-offspring correlations of kakapo telomere length.

*Parent-offspring combination*		*pairs*	*r*	p^1^
mean parent TL vs. offspring TL		26	0.295	0.144
father TL vs. offspring TL		26	-0.219	0.283
father TL vs. son TL		8	-0.067	0.875
father TL vs. daughter TL		18	-0.267	0.285
**mother TL vs. offspring TL**		**29**	**0.419**	**0.024**
**mother TL vs. son TL**		**10**	**0.767**	**0.010**
**mother TL vs. daughter TL**		19	0.302	0.208

## Discussion

### Telomere length and age

Telomere length (TL) has been found to decline with age in some species but not, or only early in life in other species [reviewed in 7]. We did not find a decline of TL with age in kakapo although the range of ages in the current study spans a minimum of 33 years. However, due to the endangered status of kakapo, we were not able to sample birds younger than 15 months. Hall et al. [13] and Pauliny et al. [16] found that in European shag (*Phalacrocorax aristotelis*), wandering albatross (*Diomedea exulans*), sand martins (*Riparia riparia*) and dunlins (*Calidris alpina*) TL decreases only early in life and stays constant thereafter. Kakapo might show a similar pattern, but a sampling of younger birds is not realizable under the current management plan. In addition, it should be noted that cross sectional studies have limited power to pick up patterns of individual TL change and may, due to selective mortality of individuals with shorter telomeres, even show a trend of increasing TL overall despite individual TL decreases [17]. However, the low mortality rate of adult kakapo [18] suggests that the lack of TL decrease with age is not a result of selective mortality. As kakapo are very long lived and the population is likely to be monitored closely in the future it would be an excellent species to illuminate the relationship between cross-sectional and longitudinal sampling in terms of telomere length over time.

### Telomere length inheritance

Telomere length (TL) is a partially inheritable trait with many loci influencing TL and/or the rate of TL change mapped to autosomes [reviewed in 5]. Heterosomal loci have occasionally been suggested to influence TL in humans [19], but cannot explain inheritance from fathers to both daughters and sons. Nordfjall et al. [10] suggested that paternal imprinting might act on autosomal genes (e.g. hTERT gene, a subunit of the telomerase enzyme) and could lead to the paternal TL inheritance found in humans. However, possible pathways leading to this imprinting are still unknown.

We found that mother TL was correlated with offspring and son TL in kakapo. This pattern mirrors findings in humans where TL is paternally inherited [10], [11] and there is a stronger correlation between father and daughter than between father and son [10]. If the same, albeit reverse, correlations exist in kakapo, this would explain why we failed to detect a correlation between mother and daughter. The weaker correlation would require a larger sample size to be detected. However in both kakapo and humans, TL is inherited from the heterogametic sex to, at least, the homogametic offspring. Therefore, we suggest that heterogamy is playing a role in TL inheritance.

Inheritance of W-chromosomal genes cannot explain TL inheritance to sons in kakapo whereas maternal imprinting could. A parental imprinting mechanism associated with heterogamy might be responsible for TL inheritance in humans and kakapo. One possibility would be a gene located on the W-chromosome in kakapo and the Y-chromosome in humans directly regulating imprinting of autosomal genes. Alternatively, autosomal genes may regulate imprinting based on expression levels of heterosomal genes. Although dosage compensation occurs for many heterosomal genes in humans and birds [20], differences in expression levels still exist in some heterosomal genes [e.g. DMRT1 (doublesex and Mab–3–related transcription factor 1), 20]. Possible targets of imprinting include telomerase itself [TERT or TERC genes, 21] or any gene involved in the regulation of telomerase and telomere capping [2].

### Sex-specific telomere length

When corrected for age, lifestyle (e.g. smoking) and other cohort factors [19], [22], TL is generally larger in female humans than in male humans [except in Amish people, 9]. Given the link between TL and cellular senescence, it is noteworthy that females also possess a greater life expectancy [4, except in Amish people, 9]. At birth, human telomeres do not differ in length between boys and girls [22], but diverge during life mediated by different rates of telomere shortening [19]. Unfortunately, we were not able to test the correlation between sex specific TL and life expectancy in kakapo, as the mortality rate in the extant kakapo population is very low [18] and few, if any, birds have reached their natural life expectancy yet [12].

Two main reasons for sex-specific telomere shortening have been suggested [4], [19], [22]: i) effects of the female hormone oestrogen and ii) the stochastic inactivation of one X-chromosome. Oestrogen may directly influence telomerase activity [22] or may be indirectly affecting TL through its antioxidant properties [4]. The stochastic inactivation of one X-chromosome in females may result in some cells harbouring an active allele favouring TL preservation and, therefore, having a selective advantage over cells harbouring an allele not favouring TL preservation. As a result, females would accumulate ‘telomere friendly’ cells throughout life and have a greater overall TL than males, who only have one allele [4].

To test these hypotheses, we measured TL in kakapo, a species with reversed heterogamy compared to humans. Given the high conservation of telomere structure and proteins/enzymes associated with TL maintenance among vertebrates [1], [23], we expect similar regulatory pathways present in human and birds. We found that telomeres were longer in male kakapo than in female kakapo, which is opposite the findings in human. However in both species, the heterogametic sex has longer telomeres.

The presence of shorter telomeres in female kakapo rather than male kakapo contradicts the suggestion that oestrogen has a conserving influence on TL. Oestrogen levels are generally higher in female birds than in male birds [24] and Cockrem et al [25] found some evidence for different estrogens levels between male and female kakapo. However, similar oestrogen levels for both sexes have been found in a number of songbirds [24], but even equal oestrogen levels in kakapo cannot explain the longer telomeres in male kakapo. It is therefore unlikely that longer telomeres are associated with higher oestrogen levels.

An inactivation of Z-chromosomal regulatory elements influencing TL, as suggested for the human X-chromosome [4], could, in theory, explain the longer telomeres in male kakapo as they are homogametic (ZZ). However, there is little or no sex-chromosome inactivation in bird species [26], ruling out this possibility for kakapo. We rather suspect that sex-specific TL is in some way associated with heterogamy: the heterogametic sex in both humans and kakapo has longer telomeres. Potentially, a gene/regulatory element could be located on the heterogametic chromosome (Y and W) that induces an accelerated loss of telomeres. As the difference in TL between the sexes are relatively small, this gene/regulatory element may not be influencing TL directly, but may act indirectly, e.g. through a metabolic pathway that induces a slightly higher rate of oxidative damage. Alternatively, a gene/regulatory element on the homogametic sex chromosome (X and Z), which is not or only partially subject to dosage compensation in the heterogametic sex, could favour TL conservation, leading to longer telomeres in the homogametic sex.

### Conclusion

It is striking that we found both reversed TL inheritance and reversed TL sex- specificity in kakapo compared to human. Although both phenomena can be explained by a variety of possibilities, the most obvious connection is heterogamy. We suggest that TL inheritance and sex specific TL is mediated by one or more pathways connected to heterogamy, possibly regulated by differential expression of heterosomal genes. However, to our knowledge human and kakapo are the only species to date for which TL inheritance and sex specificity data are available. The reversed pattern of TL regulation in these two species may be coincidence, but it may also point to a general connection between TL regulation and heterogamy. Recently, chicken has been suggested to be a valuable model system to study telomere dynamics [27] as chicken share many features of telomere dynamics with human, such as low telomerase activity in somatic cells and TL decline with age. As we do not have telomerase activity data for kakapo and there seems to be no decline of telomere length with age in kakapo it would be useful to confirm the pattern of TL inheritance we have observed in chicken, which is a more established model system of avian telomere dynamics. Our hypothesis also needs to be tested in additional species of mammals and also in species with alternative sex determination systems. Further studies should bring insight into the evolution of telomere length regulation and inheritance with respect to heterogamy and sex.

## Materials and Methods

### Ethics Statement

Approval for this work was obtained from DOC Animal Ethics Committee (blood sampling) and was done in consultation with Rūnanga o Ngāi Tahu and Kaitiaki Rūnanga Te Rūnanga o Ngāi Tahu.

The Kakapo is a highly endangered bird species native to New Zealand. It is a flightless, nocturnal lek breeder and the largest parrot alive. The current population (ca. 100 birds) is closely monitored and highly managed to increase the number of individuals while preserving genetic diversity [14]. For more information on kakapo life history see Clout [12] and references herein. Parentage was assigned by breeding monitoring [28] and mini/microsatellite analysis [14], [15], [29, Robertson unpublished data].

TL was measured in 69 kakapo using telomere restriction fragment analysis as described [30] with minor modifications. In brief, 0.5 µg DNA were restricted with *Rsa*I and *Hinf*I and resolved by constant field electrophoresis in a 0.8% agarose gel for 24 h at 50 V in TAE. DNA was denatured and processed by standard Southern blot procedure. DNA was hybridized to a digoxygenin-labelled telomere probe (TTAGGG)_4_ in Church buffer at 42°C. Signal was detected using Anti-Dig-AP Fab Fragments and CDPStar according to manufacturer's instructions (Roche). Telomere smears were analysed with ImageJ 1.38X (Java 1.6.0_02) and calibrated using a logistic model fit for the molecular weight markers. The mean signal of the whole analysis area of a blot was defined as background level. TL was calculated using the formula ∑(OD*i*•MW*i*)/∑OD*i*, with OD being the optical density at the position *i* and MW being the molecular weight at the same position *i*. A control sample was run three times on each blot to calculate the intra- and inter-gel variation (3.96% and 4.84% respectively). An example telomere blot is shown in the Figure S1. Statistical analysis was done with Minitab 15 (Minitab Inc.) and R [31]. As TL was correlated with sex, residual TL corrected for sex was used in subsequent analyses. Heritability of TL was analyzed using correlation between TL length of all offspring and mean TL of both or single parents.

## Supporting Information

Figure S1 Example of a TRF blot for kakapo. Each gel contained two molecular weight markers (M), three lanes with the reference sample (R) and ten samples (1–10).
(TIF)
