# Supplementary figures and images for: Inheritance of Telomere Length in a Bird
Source: PLoS One. 2011 Feb 22;6(2):e17199. doi: 10.1371/journal.pone.0017199 (PMC3043093; doi:10.1371/journal.pone.0017199)

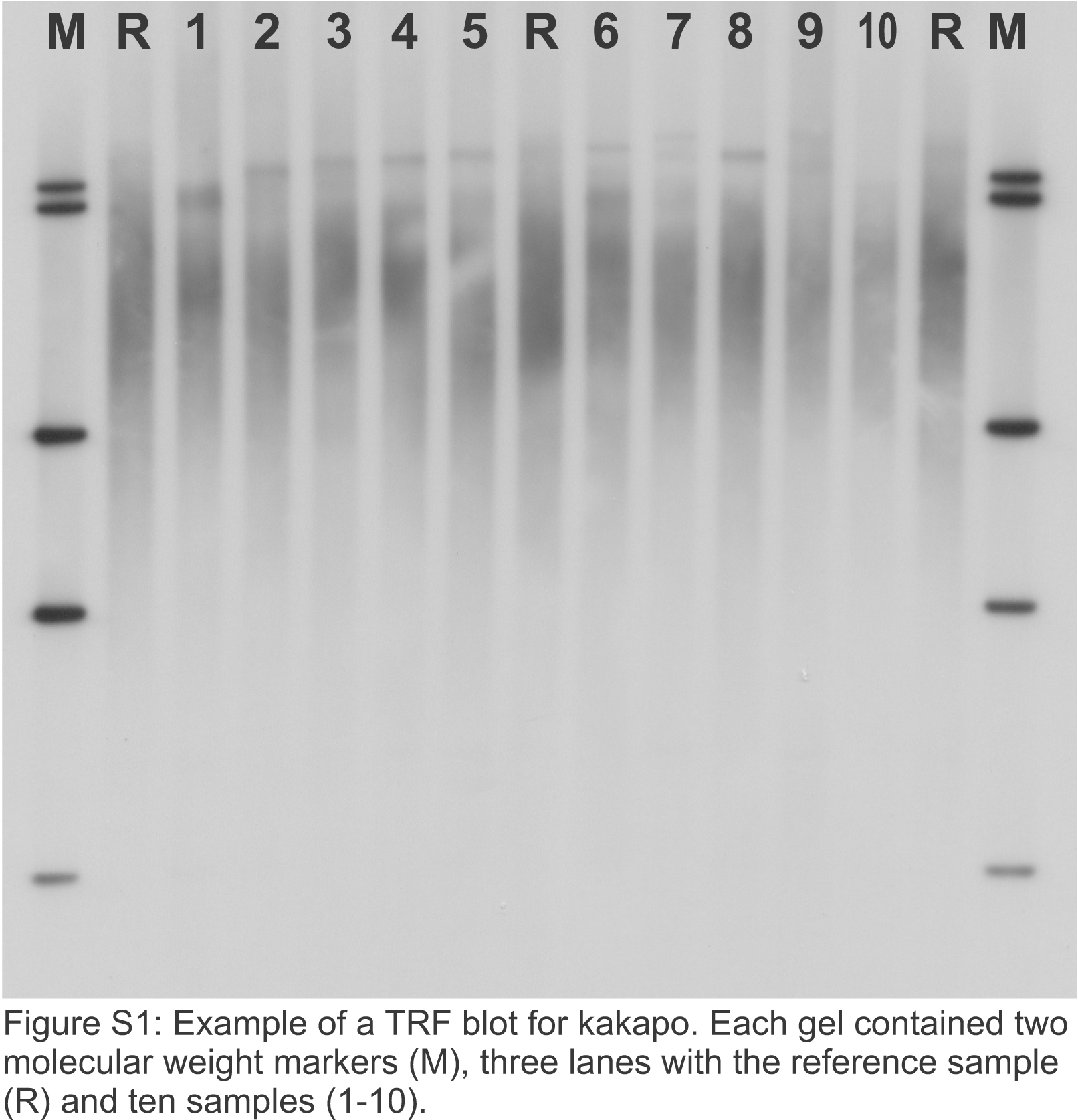

Supplement: Figure S1 — Example of a TRF blot for kakapo. Each gel contained two molecular weight markers (M), three lanes with the reference sample (R) and ten samples (1–10). (TIF) [file pone.0017199.s001.tif]
